# Supplementary material for: Influence of Microplastics on Manifestations of Experimental Chronic Colitis
Source: Toxics. 2025 Aug 21;13(8):701. doi: 10.3390/toxics13080701 (PMC12390349; doi:10.3390/toxics13080701)
Supplement: Supplementary file 1 [file toxics-13-00701-s001.zip › toxics-3787704-supplementary.pdf]

Table S1. Nucleotide sequences for real-time PCR. Abbreviations used.

Gene	Direction	Sequence (5'→3')
Actb	Forward	CCTGCCACCCAGCACAAT
	Reverse	GGGCCGGACTCGTCATAC
Cldn2	Forward	TGCGACACACAGCACAGGCATCAC
	Reverse	TCAGGAACCAGCGGCGAGTAG
Cldn4	Forward	TCGTGGGTGCTCTGGGGATGCT
	Reverse	GCGGATGACGTTGTGAGCGGTC
Cldn7	Forward	GCCTTGGTAGCATGTTCCTGGA
	Reverse	GGTACGCAGCTTTGCTTTCACTG
Muc1	Forward	GGTGACCACTTCTGCCAACT
	Reverse	TCCTTCTGAGAGCCACCACT
Muc3	Forward	TGTTCAGCTTTACTGTGTTTCAA
	Reverse	TTGCATGTCTCCTCAGGATT
Muc13	Forward	AGCATGTCCCAGCTTTCTCA
	Reverse	CCATTTGCTGCCTGAGGA
Bax	Forward	GCTGATGGCAACTTCAACTG
	Reverse	CCACCCTGGTCTTGGATC
Mki67	Forward	AGGCGAAGTGGAGCTTCTGA
	Reverse	GCTGCTGCTTCTCCTTCACTG
